# Medical Opinion and Movement

**Published:** 1908-03-21

**Authors:** 


### Medical Opinion and Movement.

ARTERIO-SCLEROSIS is probably answerable for many indefinite aches and pains and other nervous manifestations in those of advancing years, although the causal connection has received hitherto little attention. Professor Alfred Stengel has recently published a paper on the subject in the *American Journal of the Medical Sciences*. Apart from the acute symptoms which arise from sudden occlusion of a peripheral vessel, there may be numbness, tingling, a sense of heat or cold, stiffness, weakness, and pain. These symptoms may vary according to the ability of the heart to maintain the circulation in the sclerotic vessels. A tortuous and sclerotic abdominal aorta may give rise to paroxysms of pain, and Professor Stengel ascribes some cases of continuous backache in old people to this cause. Sclerosis of the aortic arch may produce paroxysms of the nature of angina pectoris, even though the coronary arteries are not involved. Cerebral symptoms, dizziness, headache, and momentary loss of consciousness, may result from sclerosis of the cerebral vessels with or without partial thrombosis or with complete thrombosis of the finer branches. It is often, however, difficult to distinguish such indefinite cerebral symptoms due to arterio-sclerosis from those caused by the chronic meningitis of old age. In fact, the two pathological conditions are often co-existent.

---

MR. W. SAMPSON HANDLEY has already won for himself a reputation by his valuable research work on the pathology of mammary cancer, particularly in regard to the spread of the disease by the lymphatics. Any operative procedure he may have to suggest is worthy, therefore, of consideration, as it is based upon a thorough knowledge of the pathology of the condition. In a recent publication he deals with the extremely painful condition of swollen brawny arm which not infrequently follows amputation of the breast for carcinoma mammæ, and he shows that this condition is due to obstruction of the lymphatic system. The permeative spread of the cancer along the lymphatics is accompanied by an obliterative process of peri-lymphatic fibrosis, which ends by the conversion of the lymphatic vessels into solid cords of fibrous tissue. In order to relieve this apparently incurable condition he has devised a method of treatment which briefly consists in laying down subcutaneous "drainpipes" in the form of silk threads. He has found that silk ligatures will remain imbedded in the tissues for years without undergoing any change. The fibrils of the silk remain free from cellular elements, and the absence of coagulation or organisation in the interior of the thread insures the retention of capillary power. He has already operated upon two cases, and the results have exceeded his expectations. Silk threads are buried subcutaneously in the arm, reaching from the wrist to the healthy loose tissue in the chest wall just below the axilla. He proposes the term "lymphangioplasty" for the operation. If subsequent experience confirms the efficacy of the operation it will be applicable to a number of other conditions due to obstructed lymphatic circulation, such as elephantiasis, chronic, "white leg," and the brawny œdema frequently associated with rheumatoid arthritis.

---

THE medical profession in this country has been slow to follow the French lead in adopting intramuscular injections as a method of prolonged mercurial treatment. Gradually, however, the evidence of its efficacy above all other methods has carried conviction, and in the meantime pain and other possible attendant inconveniences have been avoided by improvement in the technique and in the nature of the preparations injected, so that the method now appears to be gaining general approbation. The technical details of the operation are already sufficiently well known, but it is also important to have a clear idea as to the most suitable site for the injections. Frequently little more is said on this point than that the muscles of the gluteal region are usually chosen for injection. In a recent article on the subject Dr. G. Milian* lays down very definite indications. He figures each buttock as a square formed by the middle line, the outer margin, the iliac crest, and the gluteal fold. If a diagonal be drawn so as to divide this square into a superior external and an inferior internal triangle, the injections should take place entirely in the superior-external triangle. The inferior-internal triangle he designates the dangerous zone. The needle should be empty when it is introduced, so as to avoid any mercury escaping into the cellular tissue. After its introduction a few seconds should be allowed to elapse. If a vessel has been penetrated a drop of blood will rise in the needle into the syringe, and the needle must then be withdrawn and reinserted in another place. It is only by attention to such details that all mishaps and inconveniences are avoided.

---

IDIOSYNCRASIES in regard to diet are frequently encountered. A person is unable to take some one article of food. Either the stomach refuses to digest it, or when absorbed it gives rise to some such undesirable symptoms as an urticarial rash, asthma, or general malaise. When the injurious food is, say, some particular fish or vegetable, it can easily be avoided, and the evil consequences of ingestion in this way averted. But when it forms a staple article of diet, as milk or eggs, the idiosyncrasy becomes a serious inconvenience. Dr. Alfred T. Schofield has just recorded such a case, in which eggs in any form produced severe symptoms of poisoning in a boy. The boy, aged 13, was so susceptible that he was unable to take any food containing even a trace of egg in it. In the attack of intoxication there was, first of all, free secretion of saliva, the lips burned, the patient felt sick, itched, and an urticarial rash shortly broke out. He swelled all over, the eyelids and lips became puffy, the skin tight, red, and swollen, and his breathing became laboured, simulating an attack of asthma. The chief interest in the case, however, consists in the cure which Dr. Schofield was able to effect. The treatment consisted in a process of immunisation by means of minute doses of raw egg combined with calcium lactate in the form of a pill. Pills were made containing $\frac{1}{10000}$th part of a raw egg, with 2 grains of calcium lactate, and the boy was given one of these every day for a month. The dose was then gradually increased to $\frac{1}{1000}$th egg daily, and then to $\frac{1}{500}$th, and so on till by the end of six months he was taking $\frac{1}{33}$rd egg daily. Egg was then added to the food, so that in another month he was able to take $\frac{1}{8}$th of an egg, and later still he was able to consume a whole egg. The treatment, no doubt, involved much care and trouble, but the successful result was highly gratifying.

---

The administration of ergot in the treatment of hæmoptysis is a measure which has been condemned by pharmacologists. Dr. Edward Otis now defends this practice in certain selected cases. If the blood pressure is determined as a routine procedure in all sanatorium cases of tuberculosis it will be found to be lower than normal in a certain number of patients. If such a patient has hæmoptysis which does not yield to the ordinary methods, such as ice, morphia, magnesium sulphate, and rest; and if from the character of the bleeding there is further reason to suppose that it is passive and due to relaxation and transudation, Dr. Otis considers that the lowered blood pressure is an indication for ergot. In other cases in which the sphygmomanometer shows a comparatively high blood pressure, the nitrites are indicated. Dr. Otis has tested his somewhat heretical theory in a certain number of sanatorium cases of hæmoptysis associated with low blood pressure, and the results seem to have been satisfactory. But, as he himself admits, there are so many varying factors at work in the origin and causation of hæmoptysis that it is extremely difficult to estimate the effect of any one drug.

---

The common signs of death are usually well enough known to every practitioner, and there is rarely need, notwithstanding what we hear to the contrary from the Premature Burial Society, to resort to any of the more minute and exact tests which science has furnished to aid the medical attendant in declaring that life is definitely extinct in a particular patient. Nevertheless, these tests are of interest, and they ought to be known to every practitioner. Details of all of them are to be found in nearly every text-book of forensic medicine, but what has hitherto been lacking is a concise summary of their proportionate merits. This Dr. Eugene Stockis has set himself to supply in an article which is specially written for the general practitioner. Incidentally, also, he describes several less well-known tests, which are not usually detailed in ordinary text-books. The author lays stress on the uncertainty of a diagnosis founded on the posture of the dead body and on the "fœtal position" of the hands and fingers, or on such mistaken signs as that described by Collonges. This latter author finds that when the palp of a finger of the dead subject is fixed in the ear of the observer no crepitation sound can be heard, whereas in cataleptic and "apparently dead" persons a fine crepitation sound can be heard. Equally inconclusive are the signs dependent on "loss of sensibility," comprising the classical signs of Rhazes, Desgrannes, and Josais. Thanatophthalmology, or the examination of the "dead eye," gives fallacious results, in so far that the arrest of the retinal circulation and the disappearance of the optic papilla are not conclusive signs of death. The indubitable "eye sign" is the "breaking of the eyeball," as it is popularly spoken of, but this is never observed until the subject has been dead for several hours, while the signs of ocular putrefaction are equally late.

---

The absolute sign of death is, of course, commencing putrefaction, and the later tests have for their object the demonstration of early putrefactive changes in the body. Vaillant has recently drawn attention to the fact that the *x*-rays are of service in this connection. When a dead body is screened the outlines of the intestines are clearly visible under the rays, owing to the presence of sulphurous gases distending the lumen of the bowel. Becleri, however, has shown that this sign is unreliable, in that it is given in cases where a diagnosis of death is out of the question. Stockis relies chiefly on Icard's recently described "lung test," a test which is so simple and so reliable that it should be generally known. This consists in putting down into the subject's larynx a piece of filter or blotting paper impregnated with a solution of acetate of lead. If the paper is turned brown, showing the presence of sulphuretted hydrogen, it is an absolute sign of commencing putrefaction. This test succeeds within twenty-seven hours after death, and is apparently the best by means of which early putrefactive changes can be demonstrated. Lechamarzo has subjected the test to very stringent examinations in order to find whether it is possible in cases of gangrene of the lung, cancers, mediastinal tumours, phthisis, etc., for a living subject to exhale sulphuretted hydrogen, but his results have so far been negative except in one case, in which he found the paper browned after forty-eight hours' sojourn in the pharynx of a patient with extensive cancerous ulceration of the pharynx.

---

The stoppage of respiration is exceedingly difficult to prove. The candle, mirror, and water tests are fallacious. They have failed, according to the author, in several cases of syncope, and have been successful in cases of death where the expulsion of post-mortem gases has caused a flicker of the candle, obscured the image in the mirror, or rippled the surface of the water. Equally unreliable are the tests to demonstrate circulatory failure. Bonchut's classical sign (total absence of the heart sounds on auscultation for five minutes or longer) is absolutely fallacious, more especially in the case of newly-born infants, for the heart may resume its beat after a much longer period of failure. Cardiopuncture and cardiometry are of no value whatever, and the failure of the pulse is equally valueless. Vergne's test (measurement of the volume of the carotid arteries) and Laborde's (retardation of oxidation of a steel needle introduced into the deltoid muscle) are of no practical interest. Loss of translucency to light in the fingers and ears is seen in young subjects as well as in dead bodies, and the only certain test, in the author's opinion, is that of Icard (injection of flourescin solution), which is reliable, practical, and easy to apply. Icard's iodide of sodium test is less reliable. Ascarelli's test (acid reaction of the viscera) is difficult to apply and unreliable, for it demands a puncture of the liver or spleen to obtain material for the test, and it is exceedingly difficult to eliminate errors due to blood-contamination. Good general signs are, in the author's opinion, the appearance of cadaveric lividity and post-mortem staining, and Devergie's sign (patches of dessication on the skin), which is, however, late, rarely appearing before the sixteenth hour after death. Otte's sign, the so-called "explosible bleb," is quite unreliable, and has been observed in cases of syncope. Halluin has proposed a new test, which consists in dropping a one-twentieth per cent. solution of dionine into the conjunctival sac, and noting whether any reddening or achrymation results. Even in grave cases of syncope this test is reliable.

---

Boinet and Rouslacroix showed, at a recent meeting of the Marseilles Medical Society, the brain of an old man who had died in coma, accompanied with early right-sided hemiplegia and spasticity. The right hemisphere showed three small subcortical hæmorrhagic foci, and a fourth and far larger focus in the centrum ovale cutting off the parieto-occipital fibres. Such sub-thalamic hæmorrhages, according to the exhibitors, are far from common, and only a few cases are on record. They occur in elderly patients with atheromatous arteries and degenerated kidneys, and arterio-sclerosis is undoubtedly a causative factor in the ætiology. Clinically they are difficult to detect, but the exhibitors think that sufficient stress has not hitherto been laid on the heavy coma and the accompanying hemiplegia with early spasticity. A point worth attention in the differential diagnosis is the fact that there is usually marked facial paralysis, and that death ensues without any marked rise in temperature.

---

Stuart McGuire, in an analysis of the last five hundred cases of appendicitis operated upon at the St. Luke's Hospital, lays great stress on the lowered mortality due to improvements in the technique of operation, particularly in the diffuse peritonitis cases. The old method, with partial evisceration, flushing, and counter-incisions, was followed by the appalling mortality of 80 per cent.; with modern methods the mortality has been reduced to 50 per cent. and is steadily decreasing. The author recommends, in these cases of gangrenous appendix with perforation and diffuse peritonitis, that a short incision should be made over the appendix and that organ should be removed whenever possible, unless the patient's condition makes a prolongation of operation undesirable. A second incision should then be made above the pubes and a large pelvic rubber drain inserted. No sponging or washing out should be done, but the patient put back to bed as soon as possible and propped up in the Fowler position. Continuous salines should be given, the stomach washed out if vomiting persists, and sulphate of spartein given hypodermically to stimulate the heart and kidneys. In the author's hands this method of operating has had extremely good results. He has operated on eighteen cases of diffuse peritonitis with only one death. The method is certainly worth consideration, especially in cases where prolonged and extensive interference is out of the question.

---

Valentin and Vanverts communicated an interesting case of fracture of the mastoid by direct violence to the Lille Medico-Chirurgical Society at its last meeting. The patient was a woman aged twenty, who, three weeks before being seen by the communicators, had been hit by a revolver bullet which had entered behind the left ear. Three attempts at extracting the bullet had been made without success. When seen there was abundant purulent discharge from the left ear, with mastoid tenderness. The bullet was shown on the screen situated a little in front and above the point of entry. At the operation the bullet was found embedded in the posterior portion of the mastoid bone itself, which had been transversely fractured. It had been completely flattened out and had detached the dura mater. The patient rallied well from the operation, but died fifteen days later. The authors conclude from this case that radioscopy is "quite insufficient to guide the surgeon in cases of foreign bodies embedded in the cranium, since only a very expert radiographer can locate the site of the foreign body," and that in such doubtful cases by far the best method is to disregard the radioscopic findings and to follow the track of the pus.

---

An interesting discussion was initiated by Pauchet at a recent meeting of the Medico-Chirurgical Society at Lille on the question "Ought one to employ drainage after abdominal operations?" Pauchet, in introducing the subject, outlined his own routine. He has found drainage of little use in operations on the digestive tract, and argued that it produces adhesions, interferes with primary union, and leads to grave after-effects. In major operations, such as partial gastrectomy, pylorectomy, he is content with scrupulous aseptic precautions, and never drains. In cases of suppurative peritonitis and gangrenous appendicitis he employs tamponage and leaves the wound wholly open. He employs drainage in pelvic operations only when he has to deal with suppurative conditions, employing, in such cases, invariably tubes in preference to gauze tampons. Jacomet, however, thinks that "drainage should be the rule, non-drainage the exception," especially in pelvic surgery. He uses large vaginal tubes, preceded by "preliminary disinfecting drainage" by means of tampons of gauze several days before operation. Autefage also uses drainage, employing tubes in preference to gauze, which he rarely leaves in longer than forty-eight hours. The majority of those who took part in the discussion favoured drainage, especially in pelvic conditions, and above all in cases of hysterectomy for uterine fibroids.

